# Disposition and Residue Depletion of Metronidazole in Pigs and Broilers

**DOI:** 10.1038/s41598-017-07443-x

**Published:** 2017-08-03

**Authors:** Yuanhu Pan, Jiliang Yi, Bo Zhou, Shuyu Xie, Dongmei Chen, Yanfei Tao, Wei Qu, Zhenli Liu, Lingli Huang, Zonghui Yuan

**Affiliations:** 1 National Reference Laboratory of Veterinary Drug Residues and MAO Key Laboratory for Detection of Veterinary Drug Residues, Huazhong Agricultural University, Wuhan, Hubei 430070 China; 2 MOA Laboratory for Risk Assessment of Quality and Safety of Livestock and Poultry Products, Huazhong Agricultural University, Wuhan, Hubei 430070 China; 3 Hubei Collaborative Innovation Center for Animal Nutrition and Feed Safety, Huazhong Agricultural University, Wuhan, Hubei 430070 China

## Abstract

Metronidazole (MNZ) is used in veterinary medicine for the treatment of anaerobic infections and a variety of protozoal and parasitic diseases. Current study has been conducted to examine the disposition and residue depletion studies of MNZ and its main metabolites in pigs and broilers. After a single oral administration of MNZ, the concentrations of MNZ and its main metabolites in the excreta of pigs and broilers were determined by LC-MS/MS. More than 75% of the drug was recovered within 14 days, of which MNZ accounted for about 40%, MNZ-OH 20–25% and MAA less than 10%. The residue depletion study showed that MNZ was the most predominant residue in all of the tissues and could be detected in liver, kidney and muscle up to the withdrawal time of 14 days. MNZ-OH concentrations were lower than MNZ in all of the tissues, but its elimination half-life was close to MNZ. It is proposed that kidney and muscle are appropriate residue target tissues and both MNZ and its hydroxylated metabolite, MNZ-OH, should be monitored in the routine surveillance of MNZ related residues in food of animal origin.

## Introduction

Metronidazole (MNZ, 1-(hydroxyethyl)-2-methyl-5-nitroimidazole) is an antimicrobial agent which is especially used to treat anaerobic bacterial infection, protozoal and parasitic diseases^1^. Regarding its carcinogenic, mutagenic and toxic effect on host it has been banned in the EU, the United States and some of the other countries, thus no Maximum Residue Limit (MRL) has been established^2–4^. The usage of MNZ as a feed additive in food-producing animals is forbidden in China, while permitted in the treatment of histomoniasis in pigs and poultry. In a statement of China’s Ministry of Agriculture, MNZ itself is assigned as the marker residue and should not be detected in food products of animal origin^5^.

Metabolism studies have revealed that the major metabolic pathways of MNZ are oxidation of the two side-chains of the imidazole ring and glucuronide conjugation of MNZ and 1-(2-hydroxyethyl)-2-hydroxymethyl-5-nitroimidazole (MNZ-OH) in rats, dogs, rabbits and humans^6–11^. In a study on the disposition of ^14^C-MNZ in rats, four compounds, including MNZ, Glu-MNZ, MNZ-OH and 2-methyl-5-nitroimidazole-1-acetic acid (MAA) were identified as major metabolites^12^. Cybulski *et al*. studied the pharmacokinetics and whole-body autoradiography of [^3^H] MNZ in hens and quails and their results indicated that a high labelling was seen in the contents of the small and large intestines^13^. Recently, tissue distribution and residue depletion of MNZ in rainbow trout were studied by using LC-MS/MS. The results displayed that MNZ and MNZ-OH were detected in muscle up to 42 days and 21 days post treatment, respectively^14^. However, the availability of data on MNZ residue depletion in pigs and broilers is limited and according to the guidance of VICH GL46 of FDA, a comprehensive residue depletion study should contain the parent drug and its main metabolites^15^.

In this paper, a sensitive LC-MS/MS method has been developed to quantitatively determined the concentration of MNZ, MNZ-OH and MAA in the excreta of pigs and broilers following oral administration and thus to reveal the metabolism profile. Subsequently, the residue depletion of MNZ and its major metabolites in tissues of pigs and broilers were investigated. The data on the metabolism and the residue kinetics of MNZ and its metabolites in edible tissues (liver, kidney, muscle, fat etc.) of pigs and broilers should be useful for safety assessment of MNZ and offer a specific target and technical support for MNZ residue monitoring in foodstuffs of animal origin.

## Results

### Excretion of MNZ in pigs and broilers

**Figure 1:**
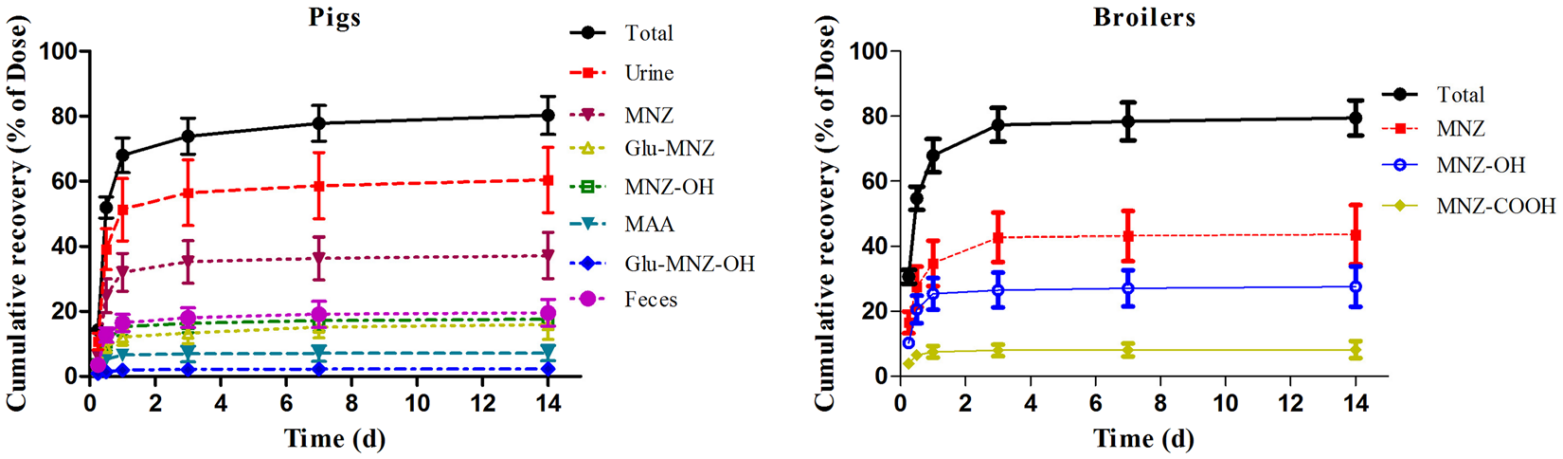
Cumulative excretion of metronidazole and its main metabolites in the excreta of pigs and broilers over 14 days after a single oral administration at 25 mg kg^−1^ b w (mean ± SD).

**Figure 2:**
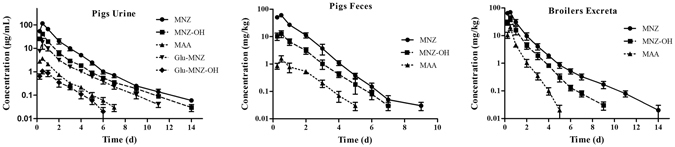
Excretion of metronidazole and it main metabolites in the excreta of pigs and broilers after a single oral administration at 25 mg kg^−1^ b w (mean ± SD).

After a single oral dosage of 25 mg kg^−1^ bw to pigs and broilers, urine and feces were collected and MNZ and it metabolites were assayed. For pigs, a total of 80.3 ± 5.9% (mean ± SD) of the administrated drug was recovered over a period of 14 days of which about 70% were collected in 24 h (Fig. 1). MNZ was excreted mainly in the urine (over 60%) while feces account for about 20% of the drug. Besides MNZ, its two oxidation metabolites (MNZ-OH and MAA) and two glucuronide conjugates were observed in the pigs urine. About 37% of the excreted drug was unchanged MNZ in the pigs urine and almost 15% was Glu-MNZ. The oxidation metabolites, MNZ-OH and MAA account for almost 18%. The plots of the concentration of MNZ and its metabolites in pigs excreta in different time courses are presented in Fig. 2. In urine, maximum concentration of the individual metabolites was observed at 12 h after dosing.

The excretion profile of broilers was similar to pigs, except that little glucuronide conjugates were found in excreta. A total of 79.4 ± 5.4% (mean + SD) of the administrated drug was recovered over a period of 14 days. Almost 45% of the excreted drug was unchanged MNZ while MNZ-OH and MAA account for 27.6% and 8.2%, respectively (Fig. 2). The maximum concentration of the individual metabolites in broilers excreta was observed at 12 h after dosing.

### Residue depletion of MNZ and its main metabolites

The mean concentrations (μg kg^−1^ ± SD) of MNZ and its metabolites in the tissues of pigs and broilers after oral administration of MNZ at the withdrawal times of 6 h, 1d, 3d, 5d, 7d and 14d are presented in Tables 1 and 2, respectively.

**Table 1:** Concentrations of MNZ, MNZ-OH and MAA in tissues of pigs after oral administration of metronidazole for 7 consecutive days (μg kg^−1^) (n = 4, Mean ± SD).

Tissues	compounds	concentration (μg kg^−1^)					
		6h	1d	3d	5d	7d	14d
Liver	MNZ	3576 ± 682	656 ± 105	196 ± 28	73 ± 15	34 ± 10	5 ± 2
	MNZ-OH	622 ± 88	152 ± 38	54 ± 15	21 ± 6	9 ± 2	ND
	MAA	58 ± 12	16 ± 4	ND	ND	ND	ND
Kidney	MNZ	4256 ± 712	731 ± 156	205 ± 54	891 ± 26	41 ± 10	8 ± 2
	MNZ-OH	569 ± 63	149 ± 24	46 ± 9	20 ± 5	11 ± 2	ND
	MAA	46 ± 9	26 ± 5	ND	ND	ND	ND
Muscle	MNZ	2289 ± 353	350 ± 81	105 ± 16	32 ± 10	18 ± 6	2 ± 1
	MNZ-OH	325 ± 62	96 ± 20	32 ± 7	15 ± 4	4 ± 1	ND
	MAA	23 ± 7	8 ± 5	ND	ND	ND	ND
Fat	MNZ	854 ± 149	126 ± 41	27 ± 6	11 ± 4	2 ± 1	ND
	MNZ-OH	203 ± 24	57 ± 12	12 ± 4	ND	ND	ND
	MAA	ND	ND	ND	ND	ND	ND
Heart	MNZ	2151 ± 386	251 ± 31	32 ± 10	15 ± 4	6 ± 3	ND
	MNZ-OH	189 ± 27	24 ± 2	ND	ND	ND	ND
	MAA	36 ± 7	6 ± 2	ND	ND	ND	ND
Lung	MNZ	3075 ± 489	151 ± 42	21 ± 6	10 ± 2	4 ± 2	ND
	MNZ-OH	492 ± 26	96 ± 25	14 ± 4	ND	ND	ND
	MAA	47 ± 5	8 ± 3	ND	ND	ND	ND
Stomach	MNZ	2885 ± 401	139 ± 41	35 ± 10	11 ± 5	3 ± 1	ND
	MNZ-OH	241 ± 61	55 ± 13	7 ± 2	ND	ND	ND
	MAA	26 ± 4	ND	ND	ND	ND	ND
Large intestine	MNZ	2611 ± 241	112 ± 29	57 ± 13	16 ± 5	3 ± 2	ND
	MNZ-OH	370 ± 41	64 ± 15	22 ± 6	6 ± 2	ND	ND
	MAA	36 ± 5	ND	ND	ND	ND	ND
Small intestine	MNZ	2972 ± 409	235 ± 23	65 ± 14	18 ± 5	4 ± 2	ND
	MNZ-OH	330 ± 34	57 ± 14	16 ± 2	4 ± 2	ND	ND
	MAA	23 ± 4	ND	ND	ND	ND	ND

ND = not detected.

**Table 2:** Concentrations of MNZ, MNZ-OH and MAA in tissues of broilers after oral administration of metronidazole for 7 consecutive days (μg kg^−1^) (n = 6, Mean ± SD).

Tissues	compounds	concentration (μg/kg)					
		6h	1d	3d	5d	7d	14d
Liver	MNZ	4672 ± 613	534 ± 75	85 ± 20	36 ± 10	20 ± 5	6 ± 2
	MNZ-OH	593 ± 102	133 ± 26	35 ± 8	15 ± 5	7 ± 2	ND
	MAA	63 ± 9	23 ± 4	ND	ND	ND	ND
Kidney	MNZ	4834 ± 495	386 ± 42	68 ± 12	28 ± 5	12 ± 3	2 ± 1
	MNZ-OH	695 ± 71	156 ± 26	45 ± 13	19 ± 4	9 ± 2	ND
	MAA	44 ± 6	18 ± 4	ND	ND	ND	ND
Muscle	MNZ	2243 ± 293	212 ± 38	68 ± 15	35 ± 12	17 ± 3	4 ± 1
	MNZ-OH	872 ± 103	201 ± 61	61 ± 15	24 ± 10	11 ± 3	ND
	MAA	31 ± 5	11 ± 3	ND	ND	ND	ND
Fat	MNZ	1565 ± 326	122 ± 21	35 ± 10	12 ± 4	5 ± 2	ND
	MNZ-OH	215 ± 55	62 ± 18	11 ± 3	ND	ND	ND
	MAA	ND	ND	ND	ND	ND	ND
Heart	MNZ	2528 ± 388	152 ± 47	32 ± 8	11 ± 4	3 ± 1	ND
	MNZ-OH	234 ± 36	73 ± 8	3 ± 1	ND	ND	ND
	MAA	37 ± 7	8 ± 2	ND	ND	ND	ND
Lung	MNZ	5417 ± 589	275 ± 42	52 ± 9	15 ± 5	4 ± 2	ND
	MNZ-OH	716 ± 98	216 ± 35	15 ± 4	ND	ND	ND
	MAA	45 ± 7	9 ± 3	ND	ND	ND	ND
Stomach	MNZ	5864 ± 614	258 ± 58	70 ± 12	10 ± 3	ND	ND
	MNZ-OH	251 ± 31	43 ± 5	2 ± 1	ND	ND	ND
	MAA	23 ± 4	ND	ND	ND	ND	ND
Large intestine	MNZ	3549 ± 438	235 ± 42	84 ± 18	22 ± 9	3 ± 2	ND
	MNZ-OH	395 ± 34	13 ± 4	ND	ND	ND	ND
	MAA	37 ± 5	ND	ND	ND	ND	ND
Small intestine	MNZ	3127 ± 364	153 ± 31	65 ± 15	16 ± 4	5 ± 1	ND
	MNZ-OH	358 ± 38	21 ± 3	ND	ND	ND	ND
	MAA	17 ± 4	ND	ND	ND	ND	ND

ND = not detected.

In pigs, MNZ, MNZ-OH and MAA can be detected in all of the tissues except that MAA was not found in fat samples at any of the time points. Six hours after the last dosing, MNZ residues in the liver, kidney and muscle tissues were at a maximum of 3.58, 4.26, and 2.29 mg kg^−1^, respectively. Moreover, the MNZ concentrations in liver and kidney were consistently higher than in other tissues and could be detected at the level close to the quantification limit (1 μg kg^−1^) at the withdrawal time of 14 days. The maximum MNZ-OH concentrations were observed in the liver samples (622 μg kg^−1^) at the 6 h time point, followed by kidney and lung tissues, and could be detected up to 7 days and 3 days, respectively, after last dosing. MAA residues were much less than MNZ in all the tissues. A peak concentration of 58 μg kg^−1^ was observed in liver 6 h post dosing and after 24 h MAA concentrations were below the quantification limit.

In broilers, a maximum MNZ concentration of 5.86 mg kg^−1^ was detected in a stomach sample at the withdrawal time of 6 h, but it was eliminated rapidly. After that, relatively high concentrations of MNZ residues were observed in liver and kidney tissues, in which MNZ residues were detected at 6 and 2 μg kg^−1^, respectively, up to 14 days post dosing. MNZ residue was also observed at 4 μg kg^−1^ in muscle tissues at the withdrawal time of 14 days. The peak MNZ-OH concentration of 872 μg kg^−1^ was observed in the muscle samples of broilers at 6 h after dosing. After 7 days, MNZ-OH residues could only be detected in liver, kidney and muscle tissues at concentrations between 2–6 μg kg^−1^. Similarly to pigs, MAA residues were much less than MNZ in tissues and at the withdrawal time of 3 days. No MAA residues were detectable in any of the broilers tissue.

**Figure 3:**
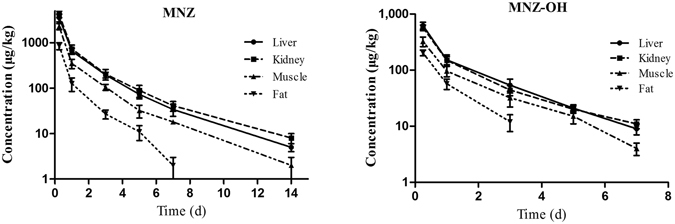
Depletion of metronidazole and its metabolites in pig tissues after 7 days of consecutive oral administration at 25 mg kg^−1^ b w (mean ± SD).

**Figure 4:**
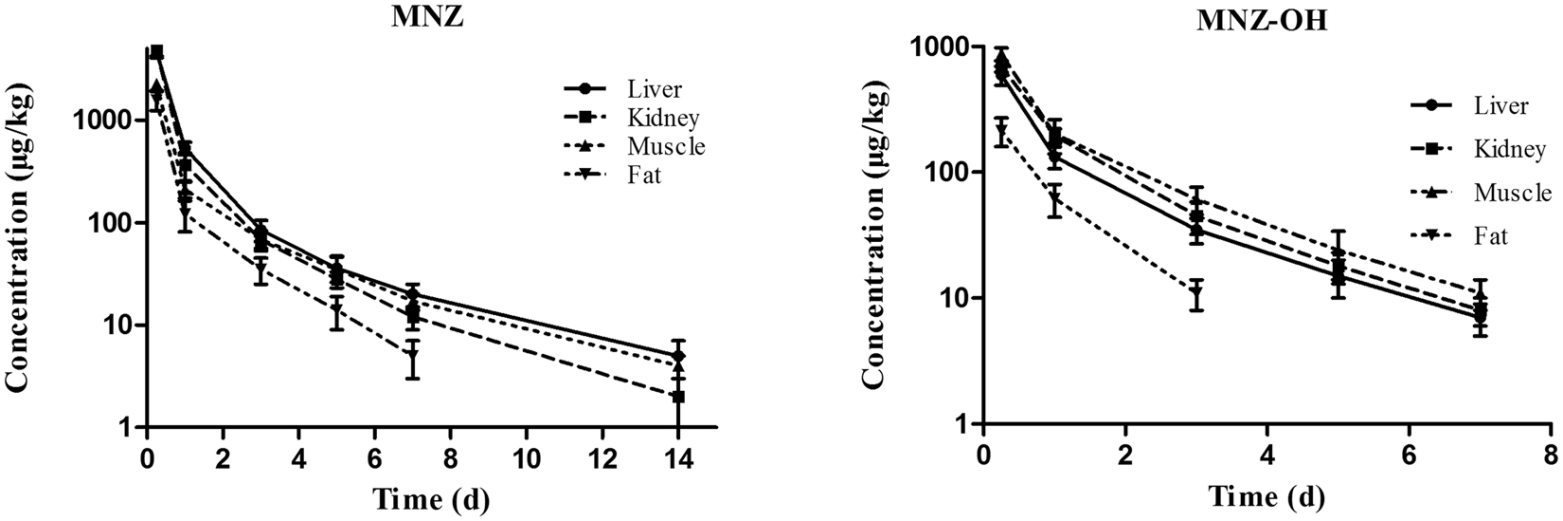
Depletion of metronidazole and its metabolites in broiler tissues after 7 days of consecutive oral administration at 25 mg kg^−1^ b w (mean ± SD).

The depletion plots of mean concentration of MNZ and MNZ-OH in the liver, kidney, muscle and fat tissues of pigs and broilers are illustrated in Figs 3 and 4. The tissue depletion profiles were characterized by a linear regression model using the log-transformed concentration of MNZ and its metabolites (ln C) against time. The last three time-point data were fit to the first-order rate equation C = C_0_ *e* ^*−kt*^, where C is the concentration of the residue, C_0_ is the initial concentration, *k* is the elimination rate constant, and the half-life of elimination (t_1/2*k*_) is calculated from the equation t_1/2*k*_ = ln 2/*k* for each tissue (Table 3). The calculated t_1/2k_ of MNZ and MNZ-OH in the tissues of pigs were similar to those of broilers. However, the elimination half-lives of MNZ and MNZ-OH in liver, kidney and muscle tissues (1.12–2.34 days) were higher than those of MNZ-OH (0.59–1.85 days). For pigs and broilers, the longest elimination half-lives of MNZ (2.23 and 2.34) were found in kidney and liver respectively, while that of MNZ-OH were found in muscle in both pigs and broilers.

**Table 3:** Elimination half-lives of MNZ and MNZOH in tissues of pigs and broilers after oral administration of metronidazole at a dose of 25 mg kg^−1^ bw day^−1^ for 7 days.

Tissue	Elimination half-lifer (days)			
	pigs		broilers	
	MNZ	MNZ-OH	MNZ	MNZ-OH
liver	2.08	1.50	2.34	1.54
kidney	2.23	1.72	1.95	1.60
muscle	1.86	1.85	2.15	1.69
fat	1.12	0.72	1.41	0.59

## Discussion

Metronidazole has been proved to be effective in the treatment of anaerobic infections and protozoal and parasitic diseases, thus it has been used legally in specific livestock species in some countries. However, MNZ has been reported as a suspected mutagen and carcinogen, so its residues in foodstuffs of animal origin might cause a hazardous effect to the consumer health^1^. Metabolism studies of MNZ in humans and various animals have shown that MNZ can be biotransformed into an oxidation product, hydroxy-metronidazole (MNZ-OH), which is thought to have comparable toxicity with the parent form. The phase II metabolites, including glucuronide and sulfate conjugates of MNZ (or MNZ-OH), were also reported. Therefore, both the parent and its metabolites should be included for the purpose of MNZ residue control.

In this study, the samples were treated with β-glucuronidase/sulfatase enzymes in order to hydrolyze the conjugated metabolites of MNZ and MNZ-OH. The results showed that the total recoveries of MNZ, MNZ-OH and MAA in the tissues of pigs and broilers were 71.4% to 93.6% with the limits of quantification set at 1 μg kg^−1^, thus proving that a reliable and sensitive LC-MS/MS method for the simultaneous determination of MNZ and its main metabolites had been achieved. Considering all MNZ metabolites detected, the percentages of products excreted over 14 days accounted for almost 80% of the drug dose. Similar findings were also reported that the total urinary excretion of MNZ in rats as well as in human subjects corresponded to 78.4% of the oral administration of the radio-labelled MNZ, pointing to the existence of other non-detectable metabolites^16^. About 80% of the excreted drug was recovered from urine, while by comparison, fecal excretion accounted for more than 15% of the excreted drug. In this context, renal excretion was considered as a primary route for elimination of MNZ and metabolites. However, the systemic bioavailability of oral MNZ in doses up to 0.8 g was reported to be approximately 100%^17, 18^. A possible explanation for the apparent discrepancy between the estimated bioavailability and the urinary recovery in this study would be that MNZ was excreted in part into the intestinal contents via biles as a glucuronide conjugate which was partly reabsorbed in the intestinal lumen^19^.

MNZ is primarily metabolized in the liver with MNZ-OH being the predominant metabolite in many species. The proportion of MNZ-OH was greater than MNZ in cumulative urine excretion from the human subjects after oral administration of MNZ^20^. In current study a considerable amount of hydroxyl-metabolite of MNZ was detected in the excreta of both pigs and broilers, but the ratio of MNZ-OH to MNZ was up to 45% in the pig’s urine as the sample collected over a time period of 14 days, while in broilers, it was more than 60%. The ratio of the other oxidation metabolite, MAA, to MNZ was less than 10% in both animals. Differences in clearance by glucuronidation were also obvious in pigs and broilers. About 15% of MNZ and 2.5% MNZ-OH were excreted in pig’s urine as a glucuronide conjugate, whereas these conjugates were not measurable in the broilers excreta. The studies together indicat a distinct species difference in metabolism and clearance of MNZ.

The residue depletion study demonstrated that MNZ was well distributed in pigs and broilers liver, kidney, heart, lung, muscle and fat tissues after oral administration for 7 consecutive days. Furthermore, the results showed that the parent drug observed at higher concentrations and was more persistent than MNZ-OH and MAA in all the animal’s tissues. MNZ could be detected at the level close to the quantification limit in liver, kidney and muscle up to 14 days post withdrawal, while MNZ-OH and MAA were present in the tissues within 7 days and 1 day respectively. Thus, MNZ is the most relevant marker residue and is consistent with a previous report^21^. It is worth noting that MNZ-OH was observed in all of the tissues after 6 h post withdrawal in which a highest MNZ-OH concentration of 872 μg kg^−1^ was present in the muscle of broilers. It was found that the ratios of MNZ-OH to MNZ in the tissues of pigs were less than those of broilers. Since MNZ-OH had been reported as having about 65% of the pharmacological activity and comparable toxicity with the parent form, the residues of MNZ-OH in the edible tissues of pigs and broilers should not be ignored^22^. The results of the current study also showed that the highest MNZ concentrations were detected in kidney followed by in liver and muscle after the withdrawal time of 1 day. The elimination half-life of MNZ in kidney was longer than in other tissues of pigs, however, it seemed that the elimination of MNZ-OH in muscle was slower than in kidney and liver. Similar trends were found in broilers, in which the MNZ-OH t_1/2*k*_ in muscle (1.69 d) was the longest in all the detected tissues, although that of MNZ was in liver tissue. Thus, kidney and muscle could be more appropriate target tissues for the effective residue control of MNZ in pigs and broilers.

The results of the current study demonstrate that MNZ is well distributed in most of the tissues of pigs and broilers after the oral administration, and is partially biotransformed into oxidation metabolites MNZ-OH and MAA, followed by glucuronide conjugation. The residue depletion studies show that the parent drug is present at higher concentrations and persists for a longer time in the tissues than other metabolites, thus it is a more suitable marker for monitoring the residue of MNZ in the edible tissues. In a CRL Guidance Paper, the hydroxymetabolites are designated as marker residue and a recommended concentration of 3 μg/kg is established for analytical methods in residue control for nitroimidazoles (Ronidazol, Dimetridazol and Metronidazol)^23^. Current results demonstrate that the recommended concentration of 3 μg/kg for MNZ-OH can not ensure the absence of the carcinogenic residue of MNZ in the edible tissues. However, a higher MNZ-OH concentration and a prolonged period of residue persistence observed in the muscle of broilers indicate that residues of MNZ-OH may also occur when MNZ is used in broilers. It is therefore proposed that both MNZ and MNZ-OH should be monitored in the routine surveillance of MNZ related residues in food of animal origin.

## Materials and Methods

### Drugs and Chemicals

The standards of MNZ, MNZ-OH, MAA and the internal standards (IS), MNZ-d_4_ (chemical and isotopic purity >98%), were sourced from Chinese Veterinary Drug Control (Beijing, China). β-Glucuronidase was obtained from Sigma Chemical Co (G0876, from *Helix pomatia*, Type H-2, ≥85000 units/mL). Cleanert PEP-2 solid-phase extraction cartridges were purchased from Agela Technologies (Agela Techonologies, Inc., China). Deionized water (Milli-Q; Millipore, Bedford, MA, USA) was used throughout the study. High-performance liquid chromatography (HPLC) grade methanol and acetonitrile was supplied by Merck Chemicals Co. (Darmstadt, Germany). All other chemicals were of analytical grade.

### Standard solutions

The MNZ, MNZ-OH, MAA and MNZ-d_4_ stock solutions of 1.0 mg mL^−1^ were prepared in acetonitrile and stored in the dark at <−18 °C. The MNZ, MNZ-OH, MAA and MNZ-d_4_ working solutions at a concentration level of 1.0 μg mL^−1^ were prepared by tenfold dilution of stock solution with water-acetonitrile (95:5, v/v) and stored at <4 °C for no longer than three months.

### Animals

Thirty-four healthy Landrace-Large white crossbred castrated male pigs (weight, 25–30 kg) were purchased from the China Breeding pig Testing Center (Wuhan, China). Forty-eight healthy white broilers (weight, 1.5–2 kg) were purchased from Wuhan China Tai broilers farm (Wuhan, China). All of the animals were allowed a 7 day acclimatisation period before the experiments commenced. A standard ration based on corn and soybean was fed twice a day and tap water was available *ad libitum*. All the animal experiment procedures were performed in accordance with the guidelines and regulations of Animal Care Center, Hubei Science and Technology Agency in China (SYXK 2013-0044) and the experimental protocols were approved by the Ethics Committee of Huazhong Agricultural University, Wuhan, China.

### Dosing and Sampling

Pigs and broilers were randomly divided into groups A, B, and C, respectively. Group A (n = 6) was fed with standard ration without MNZ. Group B (four pigs and six broilers) were orally administered with a single dose of MNZ at 25 mg kg^−1^ b w Group C (24 pigs and 36 broilers) were orally administered with MNZ at a dose of 25 mg kg^−1^ b w for 7 consecutive days. For group B, urine and feces were collected at 0–6, 6–12, 12–24 and every 24 h thereafter. All of the urinary and fecal samples were weighed and stored frozen at −20 °C. For groups A and C, one control and four medicated pigs (six medicated broilers) were anaesthetised and sacrificed at days 0.25, 1, 3, 5, 7 and 14d after the last dose. Tissue samples (liver, kidney, muscle, fat, heart, lung, stomach, large intestine and small intestine) were collected and placed in labeled plastic bags in an ice bath. All samples were assayed immediately or stored frozen at −20 °C until analysis.

### Sample preparation

All the urine and fecal samples were analyzed before and after glucuronide hydrolysis, the content of conjugates being estimated as the differences between these two assays.

#### Urine

A 2 mL urine sample was pipetted into a centrifuge tube and spiked with 100 µL of MNZ-d_4_ as an internal standard (50 ng mL^−1^). 3 mL of 0.2 mol L^−1^ acetate buffer (pH 5.2) was added and the mixture was vortex mixed for 5 min. If necessary, 40 μL of β-glucuronidase was added into the tube and the mixture was incubated at 37 °C for 12 h. The mixture was cooled to room temperature and centrifuged at 8000 rpm for 10 min. The supernatant was carefully decanted and purified using the following SPE method.

#### Feces

Aliquots of 2 g of homogenized feces were placed into a 50 mL disposable plastic centrifugal tube, followed by addition of 2 mL water plus 3 mL methanol and 100 µL of MNZ-d_4_. The mixture was extracted in an ultrasonic bath at room temperature for 10 min and centrifuged at 8000 rpm for 3 min. The supernatant was evaporated to nearly dry under reduced pressure and redissolved in 3 mL of 0.2 mol L^−1^ acetate buffer (pH 5.2). If necessary, 40 μL of β-glucuronidase was added and the mixture was incubated at 37 °C overnight. After that the sample was further processed as described below.

#### Tissues

Aliquots of 2 g tissue samples were extensively homogenized on ice in 5 mL of 0.2 mol L^−1^ acetate buffer (pH 5.2) and 100 µL of MNZ-d_4_ was added as an internal standard (50 ng g^−1^). 40 μL of β-glucuronidase was added and the mixture was incubated at 37 °C for 12 h. After the mixture was cooled to room temperature, 5 mL acetonitrile was added and the mixture was placed in an ultrasonic bath for 10 min and centrifuged at 8000 rpm for 5 min. The supernatant was transferred into a centrifugal tube and evaporated to nearly dryness under a nitrogen stream at 60 °C. The residue was redissolved in 2 mL of a solution consisting of water/methanol (95/5, v/v) and 2 mL of n-hexane was added for defatting. The aqueous phase was subjected to SPE cleanup.

### Sample Purification

The PEP-2 cartridge (60 mg, 3 mL) (Agela Technology, Inc., China) was conditioned sequentially with 3 mL of methanol and 3 mL of water. The extracted solution was loaded onto the cartridge at a flow rate of 1 mL min^−1^. The column was washed with 3 mL water and then dried by purging air at a rate of 10 mL min^−1^ for 5 min. The cartridge was then sequentially washed with 3 mL methanol and 3 mL 0.05% formic acid in methanol at a flow rate of 1 mL min^−1^. The collected elute was evaporated to dryness under a gentle stream of nitrogen at 40 °C and reconstituted in 1 mL of water-acetonitrile (95:5, v/v). The solution was filtered through a 0.22 μm syringe filter and injected into the LC-MS/MS system.

### LC-MS/MS analysis and quantification

The concentration of the analytes was measured by liquid chromatography tandem mass spectrometry (LC-MS/MS) which consists of a Surveyor Finnigan plus system with an online degasser, a Surveyor autosampler and a TSQ Quantum triple stage quadrupole mass spectrometer. Spray voltage was set at 4500 V, sheath gas and auxiliary gas were 40 and 15, respectively, and the capillary temperature was 355 °C. Product masses and collision energies were optimized by infusing the standards into the mass spectrometer. Chromatographic separation was achieved on a Shimadu VP-ODS column (150 mm × 2 mm, 5 μm) equipped with a Security-Guard C_18_ column (4 mm × 3 mm i.d.; Phenomenex). The mobile phase was a mixture of 0.1% formic acid in water (A) and 0.1% formic acid in acetonitrile (B). The gradient elution used was started from 5% B for 0.5 min, linearly increased to 90% B over 3.4 min, held for 0.5 min, and finally decreased to 5% B to re-equilibrate for 1.6 min. The flow rate was 0.4 mL min^−1^, and the column temperature was maintained at 35 °C. Detection and quantification were conducted using MRM mode to monitor precursor to production transitions for all standards. The respective MS/MS settings are presented in Table 4.

**Table 4:** LC-MS/MS parameters used for MNZ, MNZ-OH, MAA and IS quantification.

Analyte	Retention time (min)	Precursor ion (m/z)	Product ion (m/z)	Collision energy (eV)
MNZ	6.28	172.0	172.0 > 128	11
			172.0 > 82	
MNZ-OH	4.28	188.0	188.0 > 123	14
			188.0 > 126	
MAA	1.69	186.2	186.2 > 140	16
			186.2 > 169	
MNZ-d_4_	6.29	176.0	176.0 > 132	12
			176.0 > 86	

### Method validation

**Figure 5:**
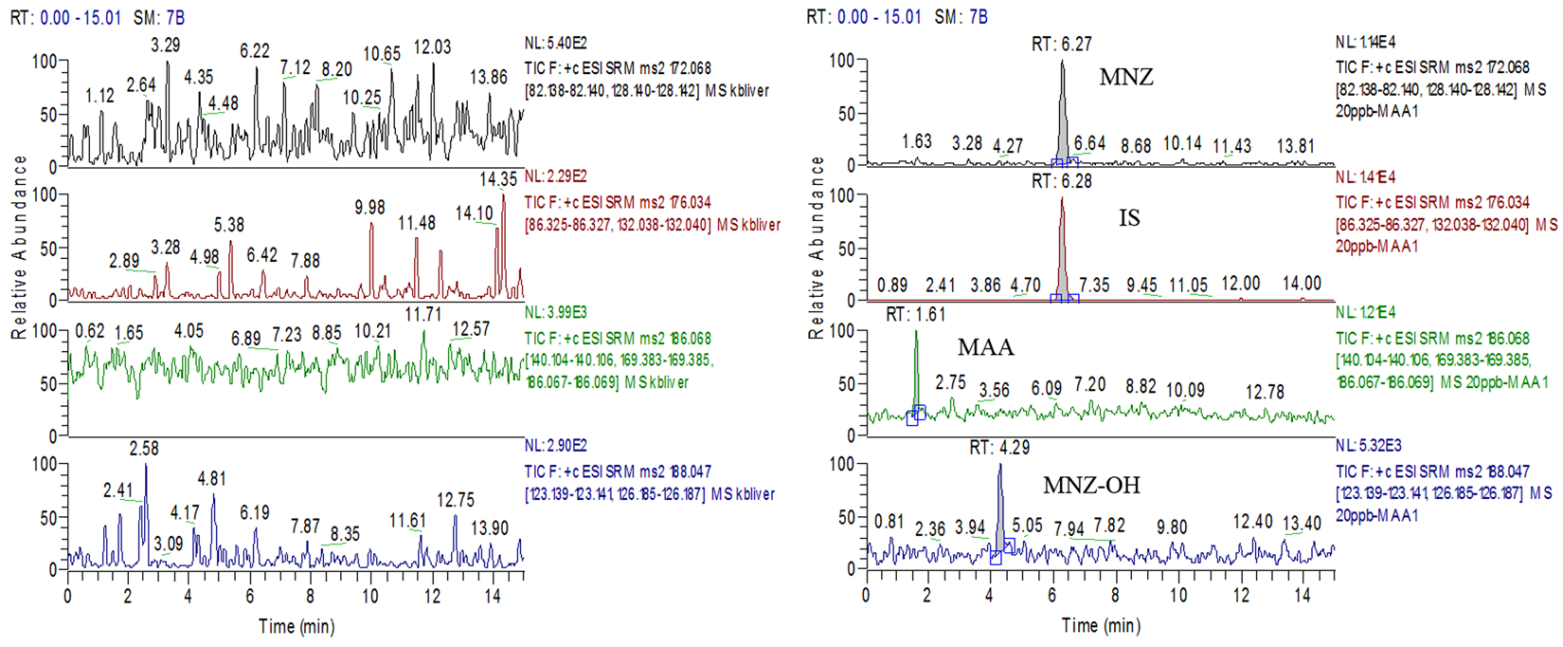
Representative chromatograms of blank liver sample (A); blank liver samples spiked with reference standards and internal standard (20 μg kg^−1^) (B)

The analysis method was validated according to EU Commission Decision 2002/657/EC criteria^24^. The specificity, matrix effects, linearity, CCα, CCβ, accuracy and precision of the method were determined by spiking blank matrices with using standard solutions containing MNZ, MNZ-OH and MAA. The specificity of the method was evaluated by the analysis of 20 control samples of liver, kidney, muscle and fat from untreated pigs and broilers. No interference from endogenous substances was observed in the retention time of the target analytes and IS. The typical chromatograms of biological specimens are shown in Fig. 5. Matrix effects on the ionization of analytes were evaluated by comparing the peak area of the standard solution with those of the matrix extract solution. The matrix-matched calibration curves were constructed by using fortified mixture working solutions in blank tissues with low (1–200 μg kg^−1^) and high (200–8000 μg kg^−1^) concentrations for MNZ and MNZ-OH and a concentration range of 5–500 μg kg^−1^ for MAA. The linear regression analysis of the calibration curves showed a good linearity with correlation coefficient above 0.9921. CCα values were calculated by the analysis of 20 blank samples of pigs and broilers, three times of the signal-noise ratio (S/N) were defined as the CCα. The CCβ was calculated by analyzing 20 blank samples spiked with the concentration at CCα, and the CCα value plus 1.64 times the corresponding standard deviation (SD) was defined as CCβ (β = 5%). The CCα and CCβ ranged from 0.5 to 1 and 1 to 5 μg kg^−1^ in the tissues of pigs and broilers, respectively, well fulfilled the sensitivity requirments of the CRL Guidance document that CCα for screening methods or CCβ for confirmatory methods should be lower than 3 μg kg^−1^ ^23^. Accuracy and precisions (intraday, interday, and within laboratory) were calculated by the determination of five aliquots of each tissue fortified at 1, 2 and 5 μg kg^−1^. The recovery of three compounds in tissues of pigs and broilers ranged from 71.4 to 93.6% and 75.2 to 92.2%, with the intraday relative RSD less than 13.2% and 12.8%, respectively. These values indicated that the established method was accurate and precise and fit for the purpose of metabolism and residue depletion studies.

### Statistical analysis

The concentration of MNZ and its metabolites were quantified by matrix match calibration curves. Descriptive statistical parameters, such as mean, SD, and CV were calculated. The residue depletion profile of MNZ in tissues of pigs and broilers was estimated by linear regression. The half-life (*t*_1/2_) of MNZ and its metabolites in different tissues during the elimination phase was calculated graphically by fitting linear regression.
